# Slow continuous dialysis treatment in septic shockcaused by acinetobacter

**DOI:** 10.1186/2197-425X-3-S1-A60

**Published:** 2015-10-01

**Authors:** S Sen, H Akdam, SB Ozturk, A Soyder

**Affiliations:** Adnan Menderes University Medical Faculty, Anesthesiology and Reanimation, Aydin, Turkey; Adnan Menderes University Medical Faculty, Nephrology, Aydin, Turkey; Adnan Menderes University Medical Faculty, Infection Diseases, Aydin, Turkey; Adnan Menderes University Medical Faculty, General Surgery, Aydin, Turkey

## Background/Purpose

Acinetobacter baumannii blood stream infection has a high mortality rate of 50-60% % for critical patients treated in ICUs [1]. The effect of continuous venovenous hemodiafiltration (CVVHDF) is described in patients with septic shock undergoing major abdominal surgery in this retrospective case series.

## Methods

Septic shock (acinetobacter baumannii) and acute renal injury (AKI) was found in 14 of the 53 patients undergoing major abdominal surgery (colon resection, Whipple). CVVHDF in 6 patients, the intermittent hemodialysis (HD) was performed in 8 patients diagnosed with septic shock.

## Results

In CVVHF group only 1 patient, in the intermittent HD group 2 patients had died. The duration of mechanical ventilation was 32.3 ± 3.8 days in the HD group, while was 23.8 ± 5 .4 day in CVVHDF group. 3 patients who died had diabetes mellitus. APACHE II scores were significantly higher in the two groups. Repetitive operation due to anastomotic leakage was made in CVVHDF group 3 patients and in HD group 4 patients.

## Discussion and Conclusion

Factors that increase mortality in patients with acinetobacter sepsis: reoperation, high APACHE II scores, mechanical ventilation, diabets mellitus and acute renal failure [1]. Continuous renal replacement therapy has the advantage of achieving a more stable haemodynamic situation and an easier volume management compared to intermittent HD. Removal of cytokines is also provided easier with CVVHDF [2]. It is concluded that mortality rate and the duration of mechanical ventilation was less in patients undergoing CVVHDF in present study.
